# Addressing Common Misconceptions in Food Allergy: A Review

**DOI:** 10.3390/children8060497

**Published:** 2021-06-11

**Authors:** Aikaterini Anagnostou

**Affiliations:** 1 Section of Immunology, Allergy and Retrovirology, Department of Pediatrics, Baylor College of Medicine, Houston, TX 77030, USA; Aikaterini.Anagnostou@bcm.edu; Tel.: +1-832-824-1319; Fax: +1-832-825-1260; 2 Section of Immunology, Allergy and Retrovirology, Department of Pediatrics, Texas Children’s Hospital, Houston, TX 77030, USA

**Keywords:** allergy, anaphylaxis, vaccines, food, epinephrine, prevention, atopic dermatitis, oral immunotherapy

## Abstract

Background: Food allergies are common, affecting 1 in 13 school children in the United States and their prevalence is increasing. Many misconceptions exist with regards to food allergy prevention, diagnosis and management. Objective: The main objective of this review is to address misconceptions with regards to food allergies and discuss the optimal, evidence-based approach for patients who carry this diagnosis. Observations: Common misconceptions in terms of food allergy prevention include beliefs that breastfeeding and delayed introduction of allergenic foods prevent the development of food allergies. In terms of diagnosis, statements such as ‘larger skin prick tests or/and higher levels of food-specific IgE can predict the severity of food-induced allergic reactions’, or ‘Tryptase is always elevated in food-induced anaphylaxis’ are inaccurate. Additionally, egg allergy is not a contraindication for receiving the influenza vaccine, food-allergy related fatalities are rare and peanut oral immunotherapy, despite reported benefits, is not a cure for food allergies. Finally, not all infants with eczema will develop food allergies and epinephrine auto-injectors may unfortunately be both unavailable and underused in food-triggered anaphylaxis. Conclusions and relevance: Healthcare professionals must be familiar with recent evidence in the food allergy field and avoid common misunderstandings that may negatively affect prevention, diagnosis and management of this chronic disease.

## 1. Introduction

Food allergies are common, affecting 6–8% of children and 10.8% of adults, and are reported to be on the rise [1,2]. The quality of life of children and their caregivers is known to be significantly affected [3]. Food-allergic patients often worry about unintentional exposures and their consequences, especially anaphylaxis and life-threatening events. Patients face multiple dietary and psychosocial restrictions (such as exclusion from peer social activities, isolation and bullying) [4,5,6,7].

Optimal care for food allergies should be based on published evidence and national or international guidelines. Unfortunately, multiple misconceptions exist regarding prevention, diagnosis, management and burden resulting from food allergies. We also live in an era when patients have access to a multitude of medical information sources, not all of which are reliable or accurate.

Studies on focus groups of parents, physicians and the general public have examined food allergy-related knowledge, attitudes and beliefs and uncovered misunderstandings and misconceptions among all the above groups [8,9,10]. For example, it was reported that physicians in different specialties presented varied approaches with regards to food allergy diagnosis and management [8,10]. They often offered conflicting advice for breastfeeding, the timing of solid food introduction and the role of eczema in food allergies [8,10].

In this review, we aim to tackle some of these common misconceptions around food allergy and clarify the optimal approach when dealing with patients who have been diagnosed with this chronic condition (see Table 1).

### 1.1. Misconception #1: Breastfeeding Is Key in Preventing Food Allergies

Breastfeeding is recommended by multiple pediatric societies around the world as the optimal nutrition for the first 6 months of life, with many benefits including a reduced risk of infection and Sudden Infant Death Syndrome during infancy, a reduced risk of cancers, improved cognitive outcomes and promotion of appropriate metabolic development through childhood [11,12,13,14]. However, its role in food allergy prevention remains controversial.

A recent report by the American Academy of Pediatrics evaluating the role of early nutritional interventions on allergic disease development stated that there are no significant benefits for exclusive breastfeeding after 3–4 months of age for preventing allergic diseases [15]. The incidence of atopic dermatitis and the risk of early childhood wheezing in the first 2 years of life is decreased however, following exclusive breast feeding, based on limited data [15]. Current evidence, cannot support a recommendation for breastfeeding in the prevention or delayed onset of IgE-mediated food allergies [15].

Some studies have suggested that breast-feeding might sensitize infants to allergenic foods at an early age, while other studies suggest this early introduction is protective [16]. Despite this controversy, breast-feeding should be encouraged for all infants, regardless of their atopy risk, due to significant long-term benefits for mother and infant [17,18]. Long-term benefits of breastfeeding include improved cognitive development, lower rates of obesity for mothers and infants and protection from a variety of chronic diseases, such as diabetes, cardiovascular problems and some forms of cancer [18]. Additionally, there are multiple reported psychological effects of breastfeeding like better mood and less stress in the mothers and improved socio-emotional development in infants [19]. For all the above reasons, exclusive breast-feeding for the first 4–6 months of life should remain the advice for promoting allergy health [17,18,19].

### 1.2. Misconception #2: Delaying Introduction of Allergenic Foods Prevents the Development of Food Allergies

In previous years, pediatric and allergy societies around the world have been advocating delaying the introduction of allergenic foods into the diet of high-risk infants after the first year of life [20]. This recommendation however, had no effect on the prevalence of food allergies, which has been steadily increasing. Research work in recent years has supported the notion that a new approach is required for food allergy prevention, especially in early life. Early introduction of foods (around the age of 6 months up to the first year of life) may prevent the development of food allergies later in life, according to recent evidence. The LEAP study has shown that peanut introduction into the diet of high-risk infants (infants with an egg allergy and/or severe eczema diagnosis), between 4–11 months of age, can prevent the subsequent development of peanut allergies [21].

Feeding guidelines for infants have seen a significant change. The National Institute of Allergy and Infectious Diseases (NIAID) published new consensus guidelines on early peanut introduction in January 2017 [22]. According to research, a minimum consumption of 2 g of peanut, at least 3 times per week, is likely needed for this intervention to be successful. Research studies have not been limited to peanut, but have also included other food allergens, in an effort to expand dietary food allergy prevention strategies to other common allergenic foods. For example, the EAT study (Enquiring About Tolerance) compared early weaning to a number of different allergenic foods versus weaning at 6 months of age in more than 1300 infants. The per-protocol analysis, which included all infants compliant with the dietary study requirements, showed a lower prevalence of food allergy in the early introduction group [23]. Additional research work also supports the notion that early introduction of allergenic foods into an infant’s diet may prevent the development of a food allergy [24,25,26,27].

Recent Australian data show that the majority of Australian parents are now compliant with the updated recommendations from the government and are successfully introducing peanut and egg into the diet of their infants by 1 year of age, which is much earlier than what was seen previously [28]. Considering that Australia has a higher rate of food allergy prevalence compared with other countries in the world, uptake of the new recommendations should result in a decrease in food allergy in the near future and studies are already underway to evaluate this.

### 1.3. Misconception #3: All Infants with Eczema Will Develop Food Allergies

Eczema has been reported as a risk factor for food allergies, but not all infants are at risk. Early onset and moderate-to-severe eczema have been mostly reported as risk factors for subsequent development of food allergies [29,30].

Martin et al. examined the link between early onset atopic dermatitis and food allergy in over 4450 infants with a confirmed food allergy [29]. All infants were evaluated for history of eczema and underwent skin prick testing for peanut, egg and sesame [29]. Those with positive testing, irrespective of wheal size, underwent an oral food challenge. The investigators reported that 1 in 5 infants with eczema were allergic to peanut, egg or sesame, compared to only 1 in 25 infants without eczema, and these results were noted to be highly significant (*p* < 0.001) [29]. The prevalence of peanut allergy was low when eczema was not present at only 0.7%. It was concluded that atopic dermatitis is a strong risk factor for IgE-mediated food allergy [29]. Infants with early onset eczema were shown to be six times more likely to have an egg allergy and 11 times more likely to have a peanut allergy by 12 months [29].

The severity of eczema also appears to play a role, with moderate and severe eczema mostly identifying infants at risk for food allergy development. In the LEAP screening study, an analysis of 834 infants revealed that sensitization to skin testing for peanut (defined as SPT 1–4 mm versus 0 mm) was associated with egg allergy and severe eczema. The investigators reported that severe eczema, egg allergy or both may be used as screening criteria to identify high-risk infants [30]. 

In summary, food allergy has been well documented in approximately one-third of children with moderate-to-severe atopic dermatitis, and an allergy workup may be performed in this selected group of patients [31].

### 1.4. Misconception #4: Larger Skin Prick Tests or/and Higher Levels of Food-Specific IgE Can Predict the Severity of Food-Induced Allergic Reactions

The diagnosis of food allergy is generally based on the history of an immediate allergic reaction following ingestion of the food and positive skin prick tests or specific IgE [32]. A study evaluating food-allergy related knowledge of primary care physicians in the United States revealed that fewer than a third felt confident in their ability to correctly interpret laboratory tests for the diagnosis of food allergy [9]. Unfortunately, commonly used diagnostic tests cannot predict the severity of the allergy or future allergic reactions [33]. The size of the diameter for skin prick testing and the levels of specific IgE to foods are helpful in establishing the diagnosis of food allergy, but do not show a correlation with allergic reaction severity [34]. In fact, anaphylaxis may be encountered in patients with both high- and low-level test results.

Component-resolved diagnostics is a developing tool for the diagnosis of food allergies. This approach aims to identify different molecular components in food allergens and their role in immediate allergic reactions [35]. Multiple food allergies have been evaluated with component testing, with certain molecular components potentially pointing towards greater allergic reaction severity, such as Ara h 2 and Ara h 6 positivity for peanut [36] and Bos d 8 positivity for milk allergy [37].

Additionally, a new testing modality, the basophil activation test, has been shown to predict severity of allergic reactions. Santos et al. report that, in a study of 124 peanut-allergic children undergoing oral food challenges, the ratio of %CD63+ basophils after stimulation with peanut and after stimulation with anti-IgE (CD63 peanut/anti-IgE) was independently associated with the severity of allergic reactions to peanut [38]. Specifically, when the above ratio was 1.3 or greater, there was an increased risk of severe reactions. The investigators concluded that basophil reactivity is associated with severity of allergic reactions to peanut [38]. Basophil activation testing is currently evaluated within the research umbrella and is not commercially available for routine testing.

In summary, currently available testing modalities cannot reliably predict the severity of food allergies, but new diagnostic tools are under investigation and may be of help in the future. In clinical practice, allergen-related factors and individual patient characteristics may be of use to physicians evaluating patients for severe allergic reactions.

### 1.5. Misconception #5: Tryptase Is Always Elevated in Food-Induced Anaphylaxis

Tryptase is a mast cell mediator, which can be elevated in anaphylaxis. Mast cells can degranulate by the cross-linking of receptor bound IgE with allergen. The presence of mast cell tryptase in serum can serve as a marker of mast cell activation, but it is important to ensure appropriate sampling conditions [39]. Paired samples are preferable to a single measurement to evaluate the extent of allergen induced mast cell activation [39,40,41]. However, it is not a marker specific for anaphylaxis [42]. It is important to remember that the diagnosis of food-induced anaphylaxis is primarily based on clinical symptoms and signs, not laboratory tests. Food-induced anaphylaxis usually presents shortly after exposure to the offending food within a 1–2 h window [43]. A raised tryptase level may be helpful when the diagnosis of anaphylaxis is uncertain clinically, but a negative result does not rule it out and false negative results are encountered frequently [44].

In episodes of food-induced anaphylaxis, tryptase may be elevated, but often it is not. In fact, in children with food-induced anaphylaxis, tryptase levels are frequently within the reported normal range [43]. Additionally, in cases of near-fatal or fatal food-induced anaphylaxis often there is no reported tryptase elevation [39,45]. For infants up to 2 years of age, however, baseline tryptase may be elevated, making interpretation of results a complicated process [46]. Peak tryptase levels are shown to be significantly higher in patients with drug-induced anaphylaxis compared with food-induced anaphylaxis [47]. An explanation for this may be that gut mucosal mast cells (who are suspected to play a key role in food-induced anaphylaxis) contain less tryptase compared to skin mast cells and may, therefore, release less tryptase into the circulation [48].

An elevated tryptase level may be the result of other causes such as mastocytosis [49], acute myelocytic leukemia, myelodysplastic syndromes, hypereosinophilic syndrome associated with the FLP1L1-PDGFRA mutation, end-stage renal failure and treatment of onchocerciasis [39]. False elevation of tryptase levels may also occur due to heterophile antibody interference during analysis [50].

In conclusion, food-induced anaphylaxis remains a clinical diagnosis. Laboratory tests such as tryptase may assist in diagnosis confirmation, but are not diagnostic per se.

### 1.6. Misconception #6: Children Allergic to Peanut Should Avoid All Tree Nuts

Children with peanut allergy are often told to avoid all tree nuts after diagnosis. Sensitization to tree nuts is frequently observed in patients with peanut allergy, but true co-allergy is observed in just over a third of patients [51,52]. Therefore, peanut-allergic individuals may also react to tree nuts or develop multiple nut allergies over time [51], but avoidance advice should be tailored to the needs of each patient rather than be used as a blanket recommendation for all.

Research studies suggest that the majority of children are able to tolerate at least some tree nuts in their diet. Recent studies such as ProNuts [53] and NUTCRACKER [54] have demonstrated that selective nut introduction is feasible and also improves quality of life. In the ProNuts study, children were diagnosed with a median of two tree nut allergies and on average were able to introduce other nuts into their diet [53]. The NUTCRACKER study revealed that cross reactivity between different tree nuts is common in patients with tree nut allergies, but over 50% of patients are only allergic to one or two nuts, with the highest rates noted for walnut and cashew [54]. Generally, the co-allergy rate for tree nuts was found to be less than 30% [54].

For selective nut introduction to work, certain steps are required. First, it is necessary to perform oral food challenges to determine allergy versus tolerance for each tree nut. Establishing the diagnosis of tree nut allergies allows for patient-tailored nut avoidance. Second, education on the correct identification of nut(s) the patient is allergic to and avoidance of the culprit nut(s) is essential for both patients and families [55]. Third, patients should receive detailed information and dietary advice on how to prevent cross-contact (also known as ‘cross-contamination’) with the index nut. Finally, after successful introduction, it is likely that the selective nuts must be consumed regularly in the diet to maintain tolerance.

### 1.7. Misconception #7: Epinephrine Auto-Injectors Are Readily Available and Frequently Used by Patients in Food Allergic Reactions

The management of food allergy includes avoidance advice, the provision of a ‘Food Allergy or Anaphylaxis Action Plan’, identification and treatment of allergic reactions and education on the use of epinephrine auto-injectors [56,57]. The use of epinephrine is the first-line treatment for food-induced anaphylaxis.

Previous reports have suggested under-prescription of epinephrine devices in patients presenting to the emergency department with anaphylaxis [58] as well as underfilling of prescriptions provided [59,60] with fewer than half of patients filling their prescription a year after discharge from the Emergency department following anaphylaxis [60].

Unfortunately, significant underuse of epinephrine auto-injectors is reported in multiple studies. In a cohort of infants 3–15 months of age who experienced anaphylaxis, epinephrine was administered in less than a third. This was the result of inability to recognize the severity of the allergic reaction, fear of the injection, uncertainty whether epinephrine was required or unavailability of the epinephrine device [61]. In a UK multi-center only 16.7% of children were reported to have used an auto-injector when experiencing anaphylaxis [62].

In the United States, Fleischer et al. reported that only one-third of severe allergic reactions were treated with epinephrine in a cohort of preschool children with a known food allergy diagnosis [63]. Additionally, in a retrospective review of 408 patients, 0–25 years old, presenting to a tertiary care pediatric emergency department with anaphylaxis, almost two-thirds had not received epinephrine prior to arrival [64].

These observations are especially worrying given that most cases of near-fatal and fatal reactions from anaphylaxis have been associated with delayed administration of epinephrine [65,66,67]. Factors associated with epinephrine underuse include high costs of prescriptions; lack of availability in patients, schools or camps; incorrect technique; complexity of diagnosing anaphylaxis; lack of knowledge about epinephrine administration; low rates of patient referrals to the allergy services and low prescription rates for those at risk [63].

In summary, epinephrine is often unavailable and underused in severe allergic reactions and significant work needs to be done in improving patient knowledge on the recognition and treatment of anaphylaxis as well as in addressing the complex psychosocial dimensions of anaphylactic emergencies [62,68].

### 1.8. Misconception #8: Egg Allergy Is a Contraindication for the Influenza Vaccine

Administration of the flu vaccine in egg-allergic children often raises concerns of risk for anaphylaxis. Research studies have demonstrated that the flu vaccine is safe for patients with egg allergy and may be given in primary care, with the usual precautions taken for any vaccination. Choosing a special egg-free vaccine, extending the usual period of observation, and administering the vaccine only in specialized medical settings are not needed and may inadvertently delay vaccination or affect vaccine uptake. Asking patients if they have an egg allergy prior to administering the influenza vaccine is also unnecessary [69,70].

In the UK, when the intranasal live attenuated influenza vaccine was administered to a highly atopic cohort of children with egg allergies, no cases of anaphylaxis were reported. This included patients with well controlled asthma or recurrent wheeze [71]. A study from Canada reinforced the above findings. Among the 68 patients with egg allergies receiving intranasal live attenuated influenza vaccine (LAIV), none suffered any allergic reactions during the hour of observation. At the 24-h follow-up, 10% of the study subjects reported non-specific adverse reactions, which did not require any treatment. The investigators concluded that the LAIV vaccine is safe for children with egg allergies, since no IgE-mediated reactions were noted after vaccination [72].

In the US, a multi-center trial reported that egg allergic children with severe allergies, including those with a history of anaphylaxis to egg ingestion, can safely receive the trivalent inactivated seasonal influenza vaccine [69]. Single-dose administration was shown to be safe in this group of patients. No allergic reactions were attributable to the vaccine, regardless of the reported level of sensitization to egg protein or other allergic comorbidity [69].

Finally, the updated Practice Parameter on Administration of the influenza vaccine to egg-allergic recipients published the following 4 recommendations in 2017: (1) Patients with an egg allergy diagnosis of any severity may receive the influenza vaccine (this data originated from 28 studies, which included a total of 4315 egg allergic patients—656 of whom had a severe egg allergy). (2) No special precautions are required for the administration of influenza vaccine to egg-allergic individuals. (3) Use of non-egg-based influenza vaccines in egg-allergic individuals (ccIIV3, RIV3, or RIV4) is not needed. (4) The live attenuated influenza vaccine may be administered to patients with egg allergies of any severity.

In contrast to the influenza vaccine, immunization for yellow fever is contraindicated in children with egg allergies, as this vaccine contains high levels of egg protein. [73,74]. The egg concentration in the vaccine may vary between 2.43–4.42 μg/mL, values that are higher than the 2 μg/mL level, which is considered the maximum safe concentration for egg-allergic individuals [73,74]. However, case series of egg-allergic patients successfully vaccinated for yellow fever with a graded dosing challenge protocol have been published [75,76].

### 1.9. Misconception #9: Oral Food Immunotherapy Is a Cure for Food Allergies

Oral food immunotherapy is a novel, active approach in the management of food allergies, allowing desensitized patients to tolerate small to medium amounts of the culprit food. The process usually takes multiple months and begins by administering a very small starting dose of the food allergen (approximately 1–25 mg of food protein) mixed into an appropriate semi-liquid vehicle (such as pudding or applesauce) [77,78]. Daily ingestion of the dose is required, followed by a gradual dose increase every 2–4 weeks until the maintenance dose is achieved (this varies between 300–5000 mg of food protein) [78,79]. The duration of up-dosing lasts 6–12 months or more depending on the maintenance dose that needs to be achieved. The large majority of patients (60–90%) are able to achieve desensitization (=increased threshold of reactivity) at the end of the intervention [78,79]. To maintain this state, a regular (usually daily) dose is required long-term.

Oral immunotherapy provides many benefits such as increased dose tolerance, protection from accidental exposures and decreased reaction severity, but is not a cure. The allergy doesn’t resolve, it is still present and allergic reactions still may occur upon large unintentional food exposures. Additionally, the intervention has to be considered in terms of both risks and benefits. Adverse reactions are frequently seen during dose escalation. The most common symptoms reported involve the gastrointestinal system (for example, oral pruritus and abdominal pain [80,81,82]). Chronic gastrointestinal symptoms are also the most common reason for stopping OIT and account for 10–36% withdrawal rates [80,81]. Dosing compliance is key for the success of this intervention and the risk of adverse events with OIT is increased when doses are missed or taken irregularly, when there is an intercurrent illness, when patients exercise within 2 h of taking their dose, or when asthma, allergic rhinitis or eczema symptoms are uncontrolled [83,84,85]. Eosinophilic esophagitis has been reported in 2.7–5.2% of individuals during OIT [86,87,88,89]. However, this adverse event appears to be reversible once the therapy is discontinued.

OIT has been investigated for cow’s milk [90,91,92,93,94,95], hen’s egg [96,97,98,99] and peanut [80,81,82,100,101,102,103]. Rates of sustained unresponsiveness are reported for up to 50% of individuals [97,104,105,106], although rates may be higher for infants and toddlers [107].

Although OIT is not a cure for food allergies, it can result in significant improvement in the quality of life for patients and their families by decreasing the fear of unintentional trace exposures, allowing expansion of their diet and decreasing severity of allergic reactions on accidental contact [108,109,110,111]. Risks and benefits should be discussed in detail with the patients as this therapy is not necessarily the optimal choice for all [112].

### 1.10. Misconception #10: Food Allergic Reactions Often Result in Fatalities

Fatalities due to anaphylaxis are a rare event with an overall case fatality rate of <0.001% [43]. A systematic review reported the incidence of food-induced fatal anaphylaxis as 1.81 per million-person year, a rate that is rarer compared with accidental death in the general population [113]. In the United Kingdom, an increase in anaphylaxis-related hospitalizations was seen over a 20-year period, but this was not linked with an increase in fatalities [114]. In Australia however, there was a reported increase in anaphylaxis fatalities (both drug- and food-induced) between 1999–2009, which may have been the result of the increasing prevalence of food allergy [115].

A recent study evaluating epidemiological data relating to food-triggered anaphylaxis and risk factors for fatalities, reported that mortality from food anaphylaxis remains low and stable. The investigators highlighted that young adults with a history of asthma and a previous diagnosis of food allergy (especially peanut, tree nuts, cow’s milk and seafood/fish allergies) were considered to be at higher risk for fatal outcomes [116]. A research study that evaluated parental knowledge related to food allergy in the United States has shown that over half the parents falsely believed younger children to be at risk of fatal outcomes, rather than adolescents, suggesting that more education is required in this area [8].

Previous reports have also reinforced the above findings. Bock et al. described 32 deaths due to food anaphylaxis. In 31 of the above cases, there was an established food allergy diagnosis, and in 94% the anaphylaxis episode was attributed to peanut or tree nuts [67]. In a UK study, food allergens were the trigger for one third of fatal cases of anaphylaxis, with over 50% associated with nuts [117,118]. A separate UK study reported that 78% of fatal cases were noted in patients with asthma, 73% were due to allergy to peanut or tree nuts and 21% were attributed to cow’s milk and seen in children less than 16 years of age [114]. Milk allergy as a cause of fatal reactions has been reported in some UK studies and is emerging as another potential cause of fatal outcomes [116]. The authors also reported a peak in food-induced fatalities in the second and third decade of life [114].

All the above case series provide crucial information in potentially identifying risk-factors and associations with fatal outcomes, but it is important to highlight that the overall number of deaths related to food induced anaphylaxis remain low and fatalities due to foods are a rare occurrence.

## 2. Conclusions

Food allergies are common and misconceptions in terms of prevention, diagnosis and management can hinder patient care and negatively impact patients and families. Healthcare professionals should be aware of frequent misconceptions and address them promptly during consultations in order to ensure evidence-based and optimal care for food-allergic patients.

## Figures and Tables

**Table 1:** Common misconceptions in food allergy.

Common Misconceptions	Current Evidence
Breastfeeding is key in preventing food allergies	Breastfeeding has many benefits in early life, but there is no evidence it can prevent food allergy development.
Delaying introduction of allergenic foods prevents the development of food allergies.	The opposite is true—early introduction of allergenic foods between 6–12 months of life may prevent food allergy development.
All infants with eczema will develop food allergies.	Not all infants with eczema are at risk for food allergy. Early onset eczema and severe eczema are mostly highlighted as risk factors.
Larger skin prick tests or/and higher levels of food-specific IgE can predict the severity of food-induced allergic reactions	This remains controversial, with most studies reporting that high levels of commonly used lab tests are not predictive of severity.
Tryptase is always elevated in food-induced anaphylaxis	Food-induced anaphylaxis is primarily a clinical, not a laboratory diagnosis. Tryptase levels may be of a help in cases of uncertainty, but they are not specific for anaphylaxis.
Children allergic to peanut should avoid all tree nuts	Emerging evidence suggests that this is not a requirement and children with peanut allergy may introduce tree nuts that they are able to tolerate into their diet.
Epinephrine auto-injectors are overprescribed and overused in food allergic reactions	The opposite is true. Epinephrine is both under-used and under-prescribed in food-allergic individuals.
Egg allergy is a contraindication for the influenza vaccine	Multiple studies report that egg-allergic patients (including those with severe egg allergy) may safely receive the influenza vaccine.
Oral food immunotherapy is a cure for food allergies	Oral food immunotherapy has many benefits, but is not a cure and will not resolve the food allergy.
Food allergic reactions often result in fatalities	Fatalities due to anaphylaxis are fortunately a rare occurrence.

## Data Availability

Not applicable.
